# Robertsonian fusion triggers recombination suppression on sex chromosomes in *Coleonyx* geckos

**DOI:** 10.1038/s41598-023-39937-2

**Published:** 2023-09-19

**Authors:** Artem Lisachov, Katerina Tishakova, Svetlana Romanenko, Lada Lisachova, Guzel Davletshina, Dmitry Prokopov, Lukáš Kratochvíl, Patricia O`Brien, Malcolm Ferguson-Smith, Pavel Borodin, Vladimir Trifonov

**Affiliations:** 1 Animal Genomics and Bioresource Research Unit (AGB Research Unit), Faculty of Science, Kasetsart University, Bangkok, 10900 Thailand; 2 Institute of Environmental and Agricultural Biology (X-BIO), University of Tyumen, Tyumen, 625003 Russia; 3 Institute of Cytology and Genetics, Russian Academy of Sciences, Siberian Branch, Novosibirsk, 630090 Russia; 4 Institute of Molecular and Cellular Biology, Russian Academy of Sciences, Siberian Branch, Novosibirsk, 630090 Russia; 5 Novosibirsk State University, Novosibirsk, 630090 Russia; 6 Department of Ecology, Faculty of Science, Charles University, 12844 Prague, Czech Republic; 7 Department of Veterinary Medicine, Cambridge Resource Centre for Comparative Genomics, University of Cambridge, Cambridge, CB3 0ES UK

**Keywords:** Chromosomes, Herpetology, Evolutionary genetics

## Abstract

The classical hypothesis proposes that the lack of recombination on sex chromosomes arises due to selection for linkage between a sex-determining locus and sexually antagonistic loci, primarily facilitated by inversions. However, cessation of recombination on sex chromosomes could be attributed also to neutral processes, connected with other chromosome rearrangements or can reflect sex-specific recombination patterns existing already before sex chromosome differentiation. Three *Coleonyx* gecko species share a complex X_1_X_1_X_2_X_2_/X_1_X_2_Y system of sex chromosomes evolved via a fusion of the Y chromosome with an autosome. We analyzed synaptonemal complexes and sequenced flow-sorted sex chromosomes to investigate the effect of chromosomal rearrangement on recombination and differentiation of these sex chromosomes. The gecko sex chromosomes evolved from syntenic regions that were also co-opted also for sex chromosomes in other reptiles. We showed that in male geckos, recombination is less prevalent in the proximal regions of chromosomes and is even further drastically reduced around the centromere of the neo-Y chromosome. We highlight that pre-existing recombination patterns and Robertsonian fusions can be responsible for the cessation of recombination on sex chromosomes and that such processes can be largely neutral.

## Introduction

Sex chromosomes have been in a multitude of shapes before they assumed a consistent form^1,2^. They can evolve from a pair of autosomes that acquired a sex-determining locus^3^. Although many sex chromosomes stay undifferentiated for a quite long evolutionary time^4^, in many lineages recombination between nascent sex chromosomes becomes suppressed around the sex-determining locus, and the non-recombining zone eventually spreads to almost the entire length of the chromosome pair^5^. Because of recombination suppression, many Y or W chromosomes diverge from their X or Z counterparts, accumulating deleterious mutations due to Muller’s ratchet^6^. In extreme cases, Y and W chromosomes may become fully heterochromatic or even lost^7,8^. Several hypotheses try to explain why there is a suppression of recombination in sex chromosomes. According to the classical model, the cessation of recombination is favored by natural selection preferring linkage between the sex-determining locus and sexually antagonistic alleles, that is, alleles that are beneficial for one sex and detrimental to the other^9^. This model is supported for example by studies on the guppy fish (*Poecilia reticulata*), a classical model species for exploring sex chromosome evolution^10,11^. However, alternative models suggest that stepwise suppression of recombination around the SDL (sex-determining locus) might occur due to neutral processes such as due to emergence of a sex-determining locus in an already ancestrally poorly recombining regions in a given sex, or mutation-induced cessation of recombination^12,13^. In this case, the presence of sexually antagonistic alleles or at least alleles beneficial only to one sex in the non-recombining region of sex chromosomes would be a consequence of recombination suppression rather than its cause. However, these models do not seem mutually exclusive and sex chromosomes in different lineages might utilize different mechanisms of recombination suppression in the heterogametic sex^14^.

Geckos (infraorder Gekkota) represent a good model group for studies onmechanisms of recombination suppression in sex chromosomes. Some gecko lineages have a putatively ancestral environmental sex determination system (ESD), while others evolved sex chromosomes of both male (XY) and female (ZW) heterogametic types from different ancestral autosomes^15–19^. The New World eublepharid genus *Coleonyx* comprises two lineages: the Central American clade containing *C. mitratus, C. elegans* and *C. nemoralis,* and the northern clade containing all the remaining species^20–22^. *C. elegans* has a complex male heterogametic sex chromosome system with X_1_X_1_X_2_X_2_ sex chromosomes in females (2n = 32) and X_1_X_2_Y sex chromosomes in males (2n = 31). Previously, we have shown that the whole chromosome probe derived from the Y chromosome of *C. elegans* hybridizes in this species with X_1_, X_2_ and Y chromosomes, suggesting that the multiple sex chromosomes evolved via afusion of the ancestral acrocentric Y with an acrocentric autosome now playing the role of a neo-X chromosome^22,23^. Alternatively, the polymorphic fused chromosomes could have been initially autosomal, and could have acquired the SDL later. The Y chromosome is the only non-acrocentric chromosome in the karyotype of *C. elegans*. It was suggested that the sex chromosomes of this species are poorly differentiated^23,24^; however, comparative genome coverage analysis using Illumina reads reveal some Y chromosome areas that are apparently degenerate^25^. These areas are homologous to parts of chicken (*Gallus gallus*) chromosomes 1, 6 and 11 (GGA1, GGA6, and GGA11 respectively). qPCR analysis reveal that the same areas are degenerate in *C. mitratus* as well*,* but its chromosomes were not cytogenetically characterized. The system of multiple sex chromosomes is missing in other eublepharid geckos and it was demonstrated that congeneric *C. brevis* possess independently evolved XX/XY sex chromosomes^21,23,25^.

In this study, we used cross-species chromosome painting with flow-sorted sex chromosome libraries of *C. elegans* to cytologically confirm their identity with the sex chromosomes of *C. mitratus* and sequenced these libraries to reveal their genomic content and elucidate the origin of the sex chromosomes of these geckos. Further, we performed immunolocalization of SYCP3, the main protein of the lateral elements of synaptonemal complexes (SC), and MLH1, a mismatch repair protein marking mature recombination nodules, to assess recombination suppression between the Y chromosome and the two X chromosomes. This method is widely used to analyze meiotic pairing and crossing over between chromosomes in vertebrates, including geckos^26^.

## Material and methods

### Specimens, DNA sequencing, assembly, annotation and COI barcoding

A group of *C. mitratus* was acquired from a commercial seller and kept according to the recommendations of Seufer et al.^27^. The species was identified by morphology as described by Klauber^28^, and species identification was further supported by similarity analysis of mitochondrial DNA sequences (DNA barcoding). All methods were performed in accordance with the relevant guidelines and regulations. All samples used in this study are listed in Table 1. DNA was extracted from muscle tissue of a male individual using the standard phenol–chloroform technique^29^. A library for low-coverage genomic sequencing was prepared using TruSeq Nano DNA Low Throughput Library Prep (Illumina), following the manufacturer's protocol. Paired-end sequencing was performed on Illumina MiSeq using ReagentKit v2 with 600-cycles (Illumina). The NGS data were deposited in the NCBI SRA database under accession number PRJNA945407. Raw Illumina reads were trimmed using Cutadapt 4.2^30^. The complete mitochondrial genome for performing DNA barcoding was assembled de novo using GetOrganelle 1.7.7.0 pipeline^31^. Mitochondrial genome was annotated using MITOS2^32^ and deposited in GenBank under accession number OQ644632. The coding sequence of the cytochrome oxidase subunit I (COI) gene was extracted from the assembly and homology search was performed using the online NCBI BLAST tool (https://blast.ncbi.nlm.nih.gov/Blast.cgi) and the BOLD database^33^.

**Table 1:** List of *C. mitratus* specimens used in this study.

Number	Sex	Age	Data obtained
1	Male	Adult	SC, mtDNA
2	Male	Adult	SC
3	Male	Subadult	SC, Cell culture
4	Male	Embryo	Cell culture
5	Male	Embryo	Cell culture
6	Male	Embryo	Cell culture
7	Female	Embryo	Cell culture

### Synaptonemal complex preparation and immunostaining

SC spreads were prepared via drying-down technique described by Peters et al.^34^. Immunofluorescence staining was performed according to the protocol by Anderson et al.^35^. Prior to immunostaining, slides were incubated in a solution of 10% PBT (PBS with 3% bovine serum albumin and 0.05% Tween 20) and 90% PBS for 45 min to reduce non-specific antibody binding. Primary antibodies included rabbit polyclonal anti-SYCP3 antibodies (1:500; Abcam, ab15093), human anticentromere antibodies (1:100; Antibodies Inc., 15-234) and mouse monoclonal anti-MLH1 antibodies (1:30, Abcam, ab14206). The slides were incubated with antibodies overnight in a humid box at 37 ℃, and then washed three times in PBS with 0.1% Tween 20 for 15 min each time. Secondary antibodies included Cy3-conjugated goat anti-rabbit (1:500; Jackson ImmunoResearch, 111-165-144), FITC-conjugated donkey anti-human (1:100; Jackson ImmunoResearch, 709-095-149) and FITC-conjugated goat anti-mouse (1:30; Jackson ImmunoResearch, 115-095-003). The slides were incubated with them for 1 h under the same conditions. After washing, the slides were mounted in Vectashield medium with DAPI (Vector Laboratories, cat No. H-1000-10) under the coverslips. Microscopic analysis and image processing were performed as described previously^36^.

### Cell cultures and mitotic chromosome preparation

Cell cultures were prepared from tissues of four *C. mitratus* embryos dissected from eggs that had been incubated for one month at 28 ℃, and from the thorax tissues of one subadult male. The cultures were established in the Laboratory of Animal Cytogenetics, Institute of Molecular and Cellular Biology, Russia, using enzymatic treatment of tissues as described previously^37,38^. The cell culture lines were deposited in the Core Facilities Center “Cryobank of cell cultures” IMCB SB RAS. Metaphase chromosome spreads were prepared from chromosome suspensions obtained from early passages of primary fibroblast cultures as described previously^39–41^.

### Flow-sorted chromosome libraries and FISH

Flow sorting of *C. elegans* chromosomes has been previously described^23,42^. Painting probes were prepared by DOP-PCR amplification of flow sorted chromosomes and labeled with biotin-dUTP and digoxigenin-dUTP (Sigma) by secondary DOP-PCR amplification as described previously^43,44^. The ribosomal DNA probe was obtained from plasmid DNA (pHr13), containing human partial 28S, full 5.8S, partial 18S ribosomal genes and two internal spacers^45^. The telomeric DNA probe was generated by PCR with oligonucleotides (TTAGGG)_5_ and (CCCTAA)_5_^46^. Labeling was performed using the «FTP-Display» DNA fragmentation kit (DNA-Display, Russia) by incorporation of biotin-dUTP and digoxigenin (dUTP). Dual-color ZooFISH was performed according to a previously published protocol^47^. Briefly, freshly made chromosome preparations were aged for 1 h at 65 °C and treated with pepsin. Chromosome denaturation was done in 70% formamide with 2 × SSC at 70 °C for 1 min. Hybridization mixture contained a hybridization buffer (50% formamide, 10% dextran sulfate, 2 × SSC), 0.2% Tween 20, 1.5 µg sonicated genomic DNA of *C. mitratus* and 0.1 µg of each labeled painting probe. Probes were denatured at 95 °C for 5 min and preannealed at 45 °C for 1 h. Hybridization was carried out at 40 °C for 48 h. The slides were analyzed with fluorescence microscope Olympus BX53 using Video-Test-FISH (VideoTestT, Saint-Petersburg, Russia) digital imaging systems.

### ChromSeq analysis

The DNA pools of the *C. elegans* chromosomes Y, X_1_, and X_2_ were sequenced as described above. The NGS data were deposited in the NCBI SRA database under accession number PRJNA945407. The resulting reads were aligned to the genome of *Eublepharis macularius* (Emac_v1.0.1)^48^ and the genome of *Anolis carolinensis* (AnoCar2.0)^49^, improved by the DNA Zoo Consortium^50,51^, using the DOPseq pipeline^52^. Synteny between reference genomes was determined using D-GENIES^53^.

### Ethical statement

All manipulations with animals were approved by the Institute of Molecular and Cellular Biology Ethics Committee (statement №01/21 from 26/01/2021).

## Results

### Species identification

The species identity of animals was confirmed by morphological traits and by DNA barcoding. The *COI* haplotype of the sequenced specimen showed 99.55% similarity with the available *C. mitratus* partial *COI* gene sequence (GenBank record ON873271), and maximum 98.92% similarity with *C. mitratus COI* sequences in the BOLD database. The assembled *C. mitratus* mitochondrial genome was deposited in GenBank under accession number OQ644632.

### Karyotypes and ZooFISH

**Figure 1:**
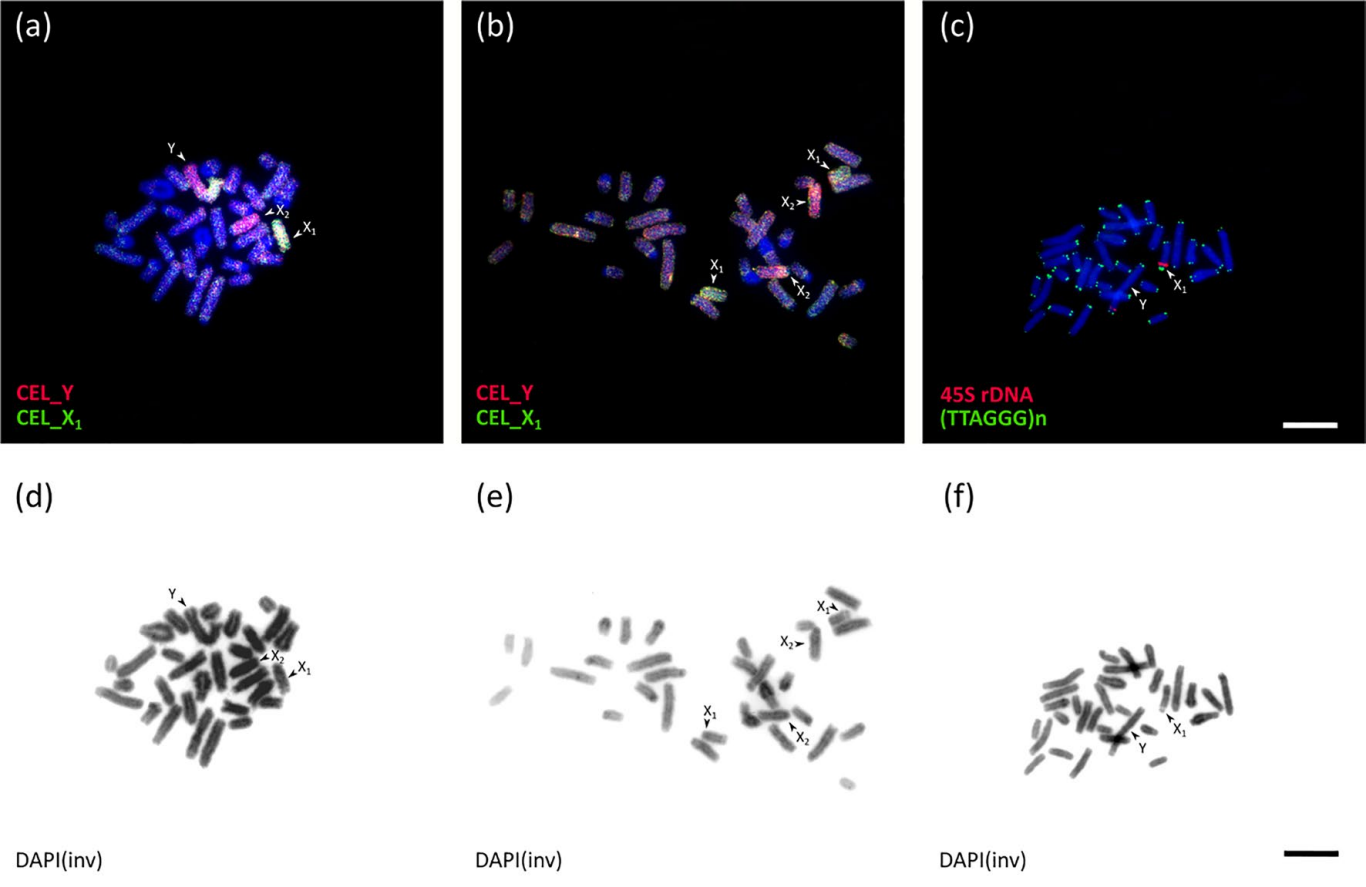
Fluorescence in situ hybridization with flow-sorted chromosome-specific probes of male *C. elegans*, 45S rDNA and telomeric probes on metaphase of *C. mitratus* (**a**–**c**) and inverted DAPI images of the same spreads (**d**–**f**). (**a**) Y chromosome specific probe (red) and X_1_ chromosome specific probe (green) on *C. mitratus* male metaphase. (**b**) Y chromosome-specific probe (red) and X_1_ chromosome specific probe (green) on *C. mitratus* female metaphase. (**c**) 45S rDNA probe (red) and telomeric probe (green) on *C. mitratus* male metaphase. Bar: 10 µm.

The mitotic karyotypes of the male and of three embryos contained 2n = 31 chromosomes, with one unpaired large metacentric chromosome. One embryo had 2n = 32 chromosomes, all acrocentric. ZooFISH with probes of *C. elegans* Y and X_1_ chromosomes painted three chromosomes in the 2n = 31 embryos (two acrocentrics and the metacentric) and four acrocentric chromosomes in the 2n = 32 embryo (Fig. 1a, b). Thus, the metacentric chromosome was identified as the Y chromosome. The terminal parts of X_1_ and Yq contained the DAPI-negative nucleolus organizer, revealed by FISH with rDNA probe. The telomeric probe hybridized to terminal parts of all chromosomes with no interstitial signals (Fig. 1c). Inverted DAPI images of the metaphases in Fig. 1a–c are presented in Fig. 1d–f, respectively.

### Synapsis and recombination of the sex chromosomes and autosomes

**Figure 2:**
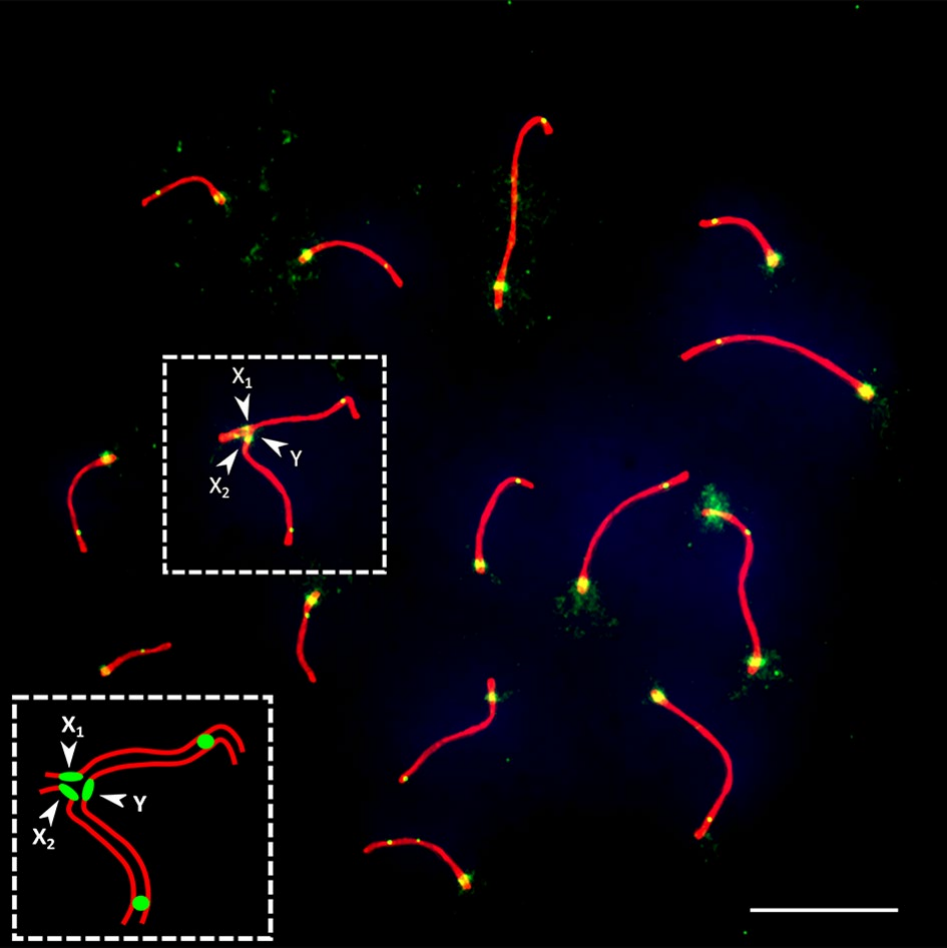
A pachytene spermatocyte of *Coleonyx mitratus*, immunolabeled with antibodies to SYCP3 (red), MLH1 (green) and centromeric proteins (green). Centromeres are indicated by larger and diffuse signals, whereas the MLH1 foci are small and round. In the upper tip of the rightmost autosomal bivalent, non-specific binding of anti-centromere antibodies in a heterochromatic area is present. Insert shows a scheme of the sex trivalent. Arrowheads show centromeres at the sex trivalent. Bar: 10 µm.

**Figure 3:**
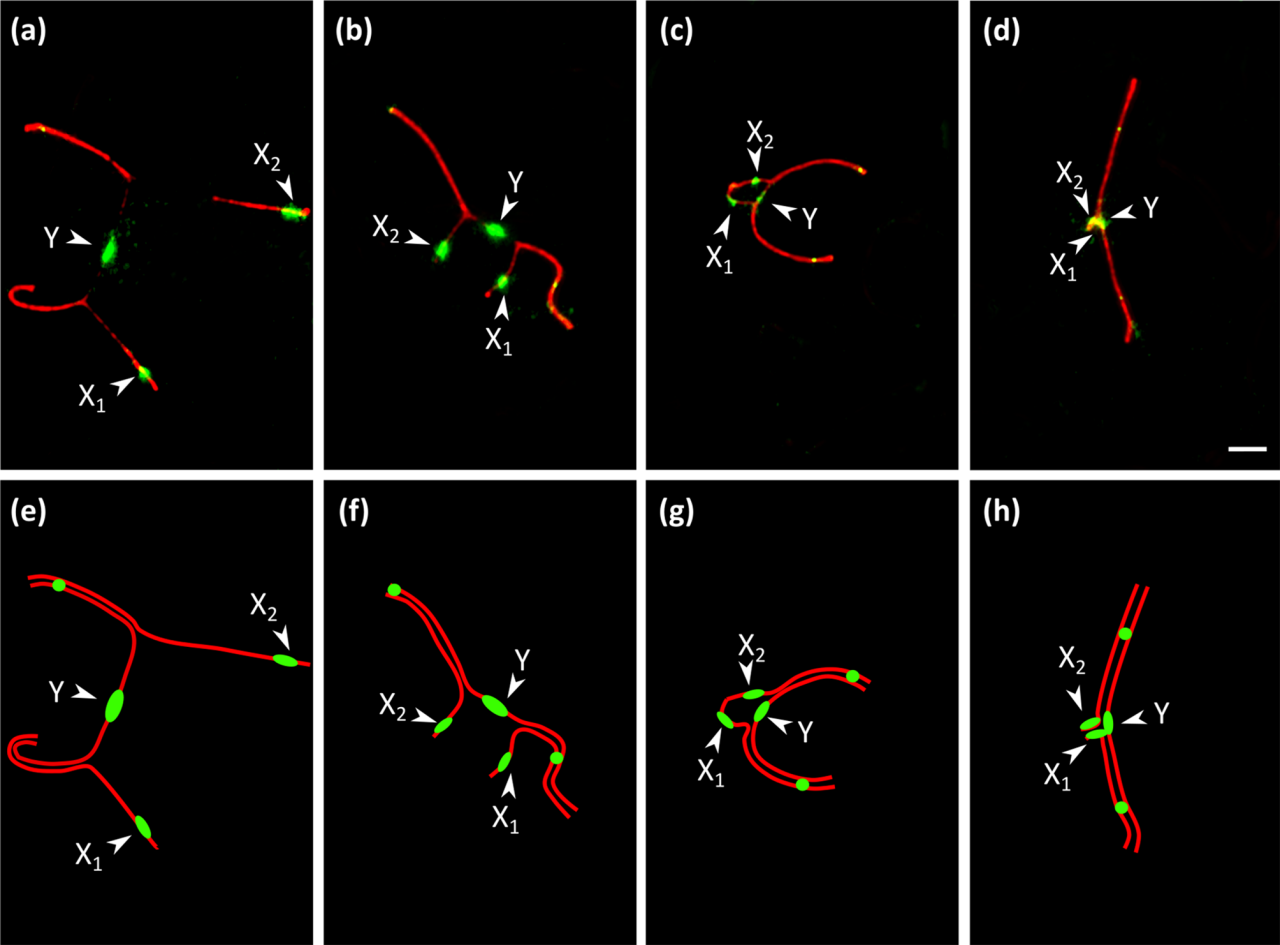
(**a**–**d**) Putative consecutive stages of sex chromosomes synapsis of *C. mitratus* after immunolocalization of SYCP3 (red), MLH1 (green) and centromeric proteins (green). Centromeres are indicated by larger and diffuse signals, whereas the MLH1 foci are small and round. (**e**–**h**) Schematic drawings of the sex trivalents. Arrowheads indicate centromeres at sex trivalents. Bar: 2 µm.

**Figure 4:**
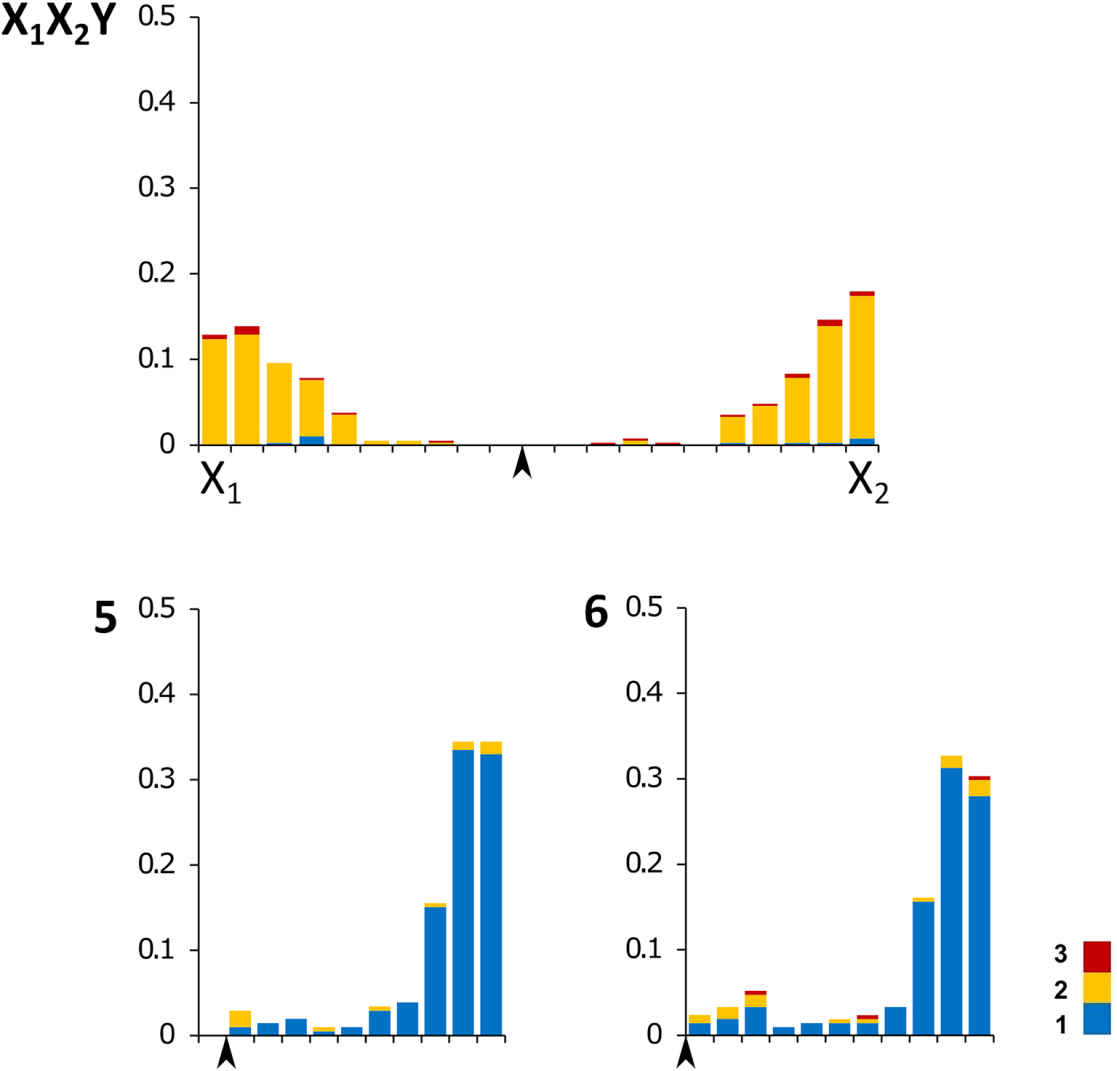
Numbers and distributions of the MLH1 foci on sex chromosome trivalents and two autosomal bivalents (chromosomes 5 and 6) in *C. mitratus.* The x-axis shows the positions of MLH1 foci along the SCs in relation to the centromere (black arrowheads). One scale division represents a segment of the average length of each SC equal to 1 µm. The y-axis shows the proportion of MLH1 foci in each interval. Different colors show SCs with different MLH1 numbers, from 1 to 3.

The SC karyotypes of the three adult males had 15 elements: 14 bivalents and the sex trivalent (Fig. 2). Recombination was studied in detail in males 1 and 2. The total SC lengths of these specimens were 186.7 ± 25.5 μm and 194.4 ± 48.9 μm (mean ± SD, 100 spreads per individual analyzed, t-test = 0.14, p = 0.89, no significant difference between individuals). In the sex trivalents, synapsis was initiated at the terminal parts. Telomeric initiation of synapsis is typical for chromosomes of vertebrates and was observed in lizards before^26,54^. The median part where the centromeric areas of the two X chromosomes fused showed delayed synapsis, indicated by incomplete synapsis when the autosomes were already fully paired, and often non-homologous pairing between the tips of the X chromosomes was observed (Fig. 3). The SC spreads of the males had 18.5 ± 1.1 and 18.7 ± 0.9 MLH1 foci (mean ± SD, 100 spreads per individual analyzed, t-test = 0.13, p = 0.9, no significant difference between individuals). Only spreads with at least one focus at each SC were considered. The sex trivalent had either two MLH1 foci, with one focus per arm (84% and 98% in two males), three foci with two foci in one arm and one in another (6% and 1% in each male, respectively), or rarely only one focus (10% and 1% in each male, respectively). The distal parts of all chromosome arms showed pronounced recombination peaks, and most acrocentric autosomal SCs also displayed minor recombination peaks close to centromeres. The proximal crossovers occurred both as single crossovers and as second crossovers in the chromosomes with MLH1 foci in both peaks. The median parts of all SCs had lower recombination than the ends. No MLH1 foci were detected in the median part of the sex trivalent (Fig. 4).

### Genetic content of the sex chromosomes

**Figure 5:**
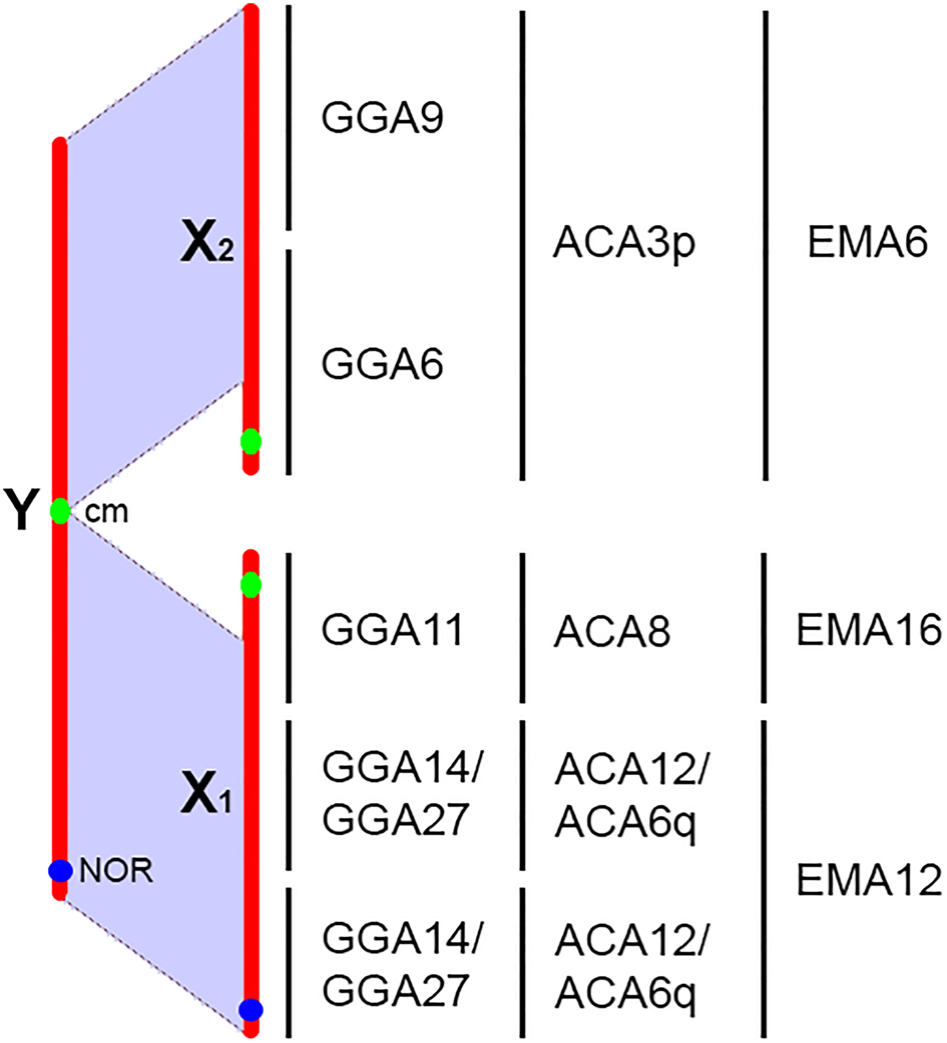
Homology between the sex chromosomes of *C. mitratus* and *C. elegans* and the chromosomes of reference species: *G. gallus* (GGA), *A. carolinensis* (ACA), *E. macularius* (EMA). Purple shading shows homology. The homologues of the proximal segments of the X chromosomes are absent in the Y chromosome due to its degeneration^24^.

According to gene content, the Y chromosome of *C. elegans* is homologous to *A. carolinensis* chromosomes 3p, 6q, 8 and 12 (ACA3p, ACA6q, ACA8, ACA12), or *E. macularius* chromosomes 6, except for the terminal 12.5 Mb (EMA6, homologous to ACA3p), chromosome 12 (EMA12, homologous to ACA6q and ACA12), and distal part of chromosome 16, except the first 10.7 Mb (EMA16, the aligned part is homologous to ACA8) (Fig. 5). According to the synteny analysis, the proximal part (approximately 12 Mb) of EMA16, not found in the Y chromosome of *C. elegans*, is homologous to a part of ACA5. The X_1_ chromosome was homologous to ACA6q, ACA8 and ACA12, or EMA12 and the distal part of EMA16, except for the first 4.9 Mb. The X_2_ chromosome library contained DNA homologous to ACA3 and ACA4q, or EMA6 and EMA5 (Fig. 5). The detailed DOPseq results are presented in Supplementary File 1.

## Discussion

### Sex chromosome synapsis and recombination

We demonstrate recombination suppression in the pericentromeric regions of the sex trivalent of *C. mitratus*, which corresponds to the earlier reported degenerate part of the Y chromosome in *C. elegans*^25^. The recombination suppression of these sex chromosomes can be attributed to several mechanisms. First, the centromere has generally a strong suppressive effect on recombination as has been demonstrated in most plants and animals examined so far^55^, although the underlying mechanisms remain unclear. Most chromosomes examined in reptiles, birds and mammals show a polarized distribution of recombination events, with peaks at chromosome ends and valleys in the middle^56,57^. In *C. mitratus,* the proximal crossover peaks in acrocentric autosomal SCs are much weaker than those in *Trapelus sanguinolentus* (Agamidae) and in birds^36,58^, indicating a strong centromeric suppression of recombination in the gecko.

Delayed synapsis, observed in the sex trivalent of *C. mitratus*, is typical for autosomal Robertsonian trivalents in many mammalian species and it is hence not special for sex chromosomes^59–61^. Metacentric SCs emerged by Robertsonian fusions in shrews, and in most mouse models, have significantly lower recombination in the centromeric region than the same chromosomes in the acrocentric form, but heterozygous metacentric trivalents do not have lower recombination in the centromeric region than homozygous metacentric bivalents^59,61,62^. Thus, we suggest that the Robertsonian heterozygosity probably has minor, if any, influence on the recombination suppression.

The total number of crossing over events on the sex chromosome trivalent in *C. mitratus* could be also the same as in the ancestral all-acrocentric states, but they can differ in distribution being localized more towards the end of the Y chromosome. Overall, the already low recombination during male meiosis near centromeres also in autosomes, and low recombination in central parts of large chromosomes could all result in the complete cessation of recombination in the median part of the Y chromosome in *C. mitratus*. In female meiosis, such large metacentric chromosomes may have more even recombination distribution due to heterochiasmy^63^. However, for the male-specific Y chromosome this means a complete cessation of recombination in the median part.

According to the classical model of sex chromosome differentiation, recombination between heterologous sex chromosomes is blocked by inversions or specific epigenetic modifications^6^. Here, the sex chromosomes probably followed another pathway: recombination in the Y chromosome was very likely suppressed due to neutral mechanistic reasons connected with the Robertsonian fusion of ancestral acrocentric Y and an acrocentric autosome, and to the general recombination patterns in males of this species^11,13^. Nevertheless, changes in gene presence and expression around the sex-determining locus before and after Robertsonian fusion can contribute to fixation of this multiple sex chromosome system in the common ancestor of *C. elegans* and *C. mitratus*.

### Sex chromosome contents and homology

The karyotype of *C. mitratus* was identical to the previously known karyotype of *C. elegans*^23^, in accordance with their sex chromosome identity^25^ and similarity of flow-sorted karyotypes^42^. The degenerate parts of *C. mitratus* and *C. elegans* Y chromosomes are homologous to a part of chicken chromosome 11 (GGA11, homologous to ACA8), small proximal part of chromosome 6 (GGA6), and small median part of chromosome 1 (GGA1) as was shown in the previous study based on a different technique, i.e. the comparison of coverage between sexes^25^. In *Podarcis muralis*, both latter parts are homologous to the proximal part of chromosome 5 (homologous to ACA3p).

By sequencing the chromosome-specific DNA libraries of *C. elegans*, we were able to reveal the remaining fragments that did not experience degeneration and were therefore not detected by the previous coverage analysis. We assigned these fragments to chromosomes X_1_ and X_2_. Moreover, data on Y chromosome degeneration, synapsis data and crossover map allow us to reconstruct the orientation of the syntenic regions inside the sex chromosomes. First, the synaptic configuration of the sex trivalent shows that the formation of the Y chromosome resulted from a centromere-to-centromere, but not centromere-to-telomere, or telomere-to-telomere fusion. Second, the suppression of recombination in the median part of the trivalent shows that the Y-degenerate parts are in the proximal segments of the X chromosomes. The schematic reconstruction is shown in Fig. 5. The only uncertain element is the order of the ACA6q-homologous and ACA12-homologous fragments.

The association between the homolog of ACA8 and a part of ACA5, found in EMA16, was not detected in *C. elegans.* This may be caused by either an *E. macularius-*specific translocation or a genome assembly error. The terminal part of EMA6, showing very low coverage with Y chromosome reads, probably corresponds to the degenerate part. The degenerate part of the ACA8-homologous segment was not detected by DOPseq either due to low coverage in this region or lower level of degeneration. The presence of DNA homologous to EMA5 (ACA4q) in the X_2_ probe indicates contamination with a similar-size autosome.

The recruitment of autosomes for the role of sex chromosomes is non-random, and some genomic regions are more frequently involved in sex chromosome formation due to their genetic content^64^. Several squamate species have been found to use the synthetic regions found on the *C. elegans* and *C. mitratus* sex chromosomes as part of their sex chromosomes. The involvement of ACA3p/GGA6 and ACA8/GGA11 was discussed previously^25^. ACA6q/GGA27 is a conserved element of ZZ/ZW sex chromosomes of caenophidian snakes^65^ and XX/XY sex chromosomes of *Python bivittatus*^66^. The syntenic block homologous to ACA12/GGA14 has been reported as part of the ZZ/ZW sex chromosomes of the marbled gecko (*Christinus marmoratus*)^67^, and as the pseudoautosomal part of the XX/XY sex chromosomes of the brown anole (*Norops sagrei*)^68^.

## Conclusion

In the current study we revealed a possible mechanism of recombination suppression in the sex chromosomes of *C. mitratus* via Robertsonian fusion of sex chromosomes and reduced overall recombination in pericentromeric regions in male meiosis, as reported for other cases in which Robertsonian translocations are present. This finding is consistent with the neutral hypotheses of sex chromosome recombination suppression^12,13^, although further tests of gene content and gene expression are needed to determine the involvement of sexually antagonistic selection. Accurate fine-scale assemblies of the three sex chromosomes of these geckos and a study of female meiosis are required to understand the recombination between them in further detail. The identification of all syntenic regions involved in the formation of this complex sex chromosome system, as performed in this study, will help to understand the possibility of parallel co-option of different syntenic regions during sex chromosome formation in vertebrates.

### Supplementary Information

Supplementary Information.

## Data Availability

All raw sequence data are deposited in the NCBI SRA database under accession number PRJNA945407.
